# In silico identification of a novel Cdc2‐like kinase 2 (CLK2) inhibitor in triple negative breast cancer

**DOI:** 10.1002/pro.5004

**Published:** 2024-05-09

**Authors:** Cheng‐Chiao Huang, Chia‐Ming Hsu, Min‐Wu Chao, Kai‐Cheng Hsu, Tony Eight Lin, Shih‐Chung Yen, Huang‐Ju Tu, Shiow‐Lin Pan

**Affiliations:** ^1^ Ph.D. Program for Cancer Molecular Biology and Drug Discovery, College of Medical Science and Technology, Taipei Medical University and Academia Sinica Taipei Taiwan; ^2^ Division of General Surgery, Department of Surgery Taipei Medical University Hospital Taipei Taiwan; ^3^ Graduate Institute of Cancer Biology and Drug Discovery, College of Medical Science and Technology, Taipei Medical University Taipei Taiwan; ^4^ School of Medicine, College of Medicine, National Sun Yat‐sen University Kaohsiung Taiwan; ^5^ Institute of Biopharmaceutical Sciences, College of Medicine, National Sun Yat‐sen University Kaohsiung Taiwan; ^6^ The Doctoral Program of Clinical and Experimental Medicine, College of Medicine, National Sun Yat‐sen University Kaohsiung Taiwan; ^7^ Ph.D. Program in Drug Discovery and Development Industry College of Pharmacy, Taipei Medical University Taipei Taiwan; ^8^ TMU Research Center of Cancer Translational Medicine, Taipei Medical University Taipei Taiwan; ^9^ TMU Research Center of Drug Discovery, Taipei Medical University Taipei Taiwan; ^10^ Warshel Institute for Computational Biology, The Chinese University of Hong Kong (Shenzhen) Shenzhen Guangdong China

**Keywords:** CLK2, drug discovery, kinase inhibitor, structure‐based virtual screening, triple‐negative breast cancer

## Abstract

Dysregulation of RNA splicing processes is intricately linked to tumorigenesis in various cancers, especially breast cancer. Cdc2‐like kinase 2 (CLK2), an oncogenic RNA‐splicing kinase pivotal in breast cancer, plays a significant role, particularly in the context of triple‐negative breast cancer (TNBC), a subtype marked by substantial medical challenges due to its low survival rates. In this study, we employed a structure‐based virtual screening (SBVS) method to identify potential CLK2 inhibitors with novel chemical structures for treating TNBC. Compound 670551 emerged as a novel CLK2 inhibitor with a 50% inhibitory concentration (IC_50_) value of 619.7 nM. Importantly, Compound 670551 exhibited high selectivity for CLK2 over other protein kinases. Functionally, this compound significantly reduced the survival and proliferation of TNBC cells. Results from a cell‐based assay demonstrated that this inhibitor led to a decrease in RNA splicing proteins, such as SRSF4 and SRSF6, resulting in cell apoptosis. In summary, we identified a novel CLK2 inhibitor as a promising potential treatment for TNBC therapy.

## 1 INTRODUCTION

Breast cancer (BC) is the most common cancer among women globally, and its incidence continues to rise annually. BC can be categorized based on the expression status of the estrogen receptor (ER), progesterone receptor (PR), and human epidermal growth receptor 2 (HER2). Remarkable progress has been made in developing targeted therapies for these biomarkers, significantly improving both patient survival rates and progression‐free survival. However, tumors in patients without ER, PR, and HER2 expression, commonly referred to triple‐negative breast cancer (TNBC), exhibit higher aggressiveness and metastatic potential compared to other BC subtypes (Ensenyat‐Mendez et al., 2021). Owing to the absence of these receptors, TNBC does not respond to conventional targeted therapies, rendering it particularly challenging to manage. This is most evident in metastatic TNBC, where the limited availability of effective treatments leads to a 5‐year survival rate of only 10.81% (Hsu et al., 2022). Therefore, TNBC presents a pressing unmet medical challenge, requiring the discovery of novel targets to address this disease.

One of the primary challenges in treating TNBC is its heterogeneity, which characterized by a wide range of histological, genetic, and molecular profiles, alongside differing treatment responses (Other‐Gee Pohl & Myant, 2022). This variability is partly due to alternative splicing (AS) events, which contribute to the proteomic diversity of TNBC cells and affect cancer hallmarks such as proliferative signaling, evasion of growth suppression, and angiogenesis (Sudhakaran et al., 2023). AS is integral to numerous physiological and pathological processes, approximately 95% of human genes undergo AS, generating diverse transcripts depending on the tissue or cellular conditions. The aberrant regulation of AS usually results in the rapid expression of gene products and mutations that contribute to cancer development. Analysis of a substantial cohort of cancer patients has revealed an increased prevalence of AS events in cancerous tissues, with over 30% more occurrences compared to normal samples (Kahles et al., 2018). Specifically, abnormal AS is significantly associated with key processes such as cell adhesion, the cell cycle, and immune response modulation in TNBC (Yu et al., 2020).

One key factor in regulating AS event is splicing factor (SF) proteins, which includes small nuclear ribonucleoproteins (snRNPs), heterogeneous nuclear ribonucleoproteins (hnRNPs), and Ser/Arg‐rich (SR) proteins. Aberrant SFs, including those with abnormal expressions, functions, or post‐translational modifications, were demonstrated to contribute to the proliferation, migration, apoptosis, and pathological processes of cancers, such as liver, breast, colorectal, lung, glioblastoma, prostate, and kidney cancers (Zheng et al., 2020). Furthermore, studies have identified a correlation between AS events and the expression levels of SFs in TNBC, indicating the potential of SFs as prognostic biomarkers for assessing cancer invasiveness and patient survival rates (Wu et al., 2020). Therefore, targeting SR proteins and SR‐related kinases is a novel therapeutic strategy for cancer treatment (Tang et al., 2022).

Cdc2‐like kinase 2 (CLK2) is a nuclear dual‐specificity protein kinase that acts on serine/threonine and tyrosine‐containing substrates, such as SR proteins (Duncan et al., 1998). Catalytically active CLK2 can regulate pre‐mRNA AS and is closely associated with various diseases, particularly in the pathogenesis of cancers (Blackie & Foley, 2022). In TNBC, the aberrant regulation of AS by CLK2 emerges as a critical factor influencing tumor progression. Study has shown that genetic silencing of CLK2 in TNBC significantly reduces tumorigenesis (Yoshida et al., 2015). Previous research has demonstrated that pharmacological inhibition of CLK2 caused splicing alterations of S6K pre‐mRNA in TNBC, underscoring a strong correlation between CLK2 activity and AS events (Araki et al., 2015). The CLK inhibitor T‐025, which is notably effective against CLK2, has shown substantial antitumor effects in TNBC, especially in aggressive MYC‐driven BC (Iwai et al., 2018). Moreover, a dual CLK2/TKK inhibitor exhibited pronounced antiproliferative effects in various TNBC cell lines while exerting minimal impact on luminal breast cancer cells. This distinction highlights the potential of the CLK2 inhibitor as a specialized therapeutic approach for TNBC (Riggs et al., 2017).

In this study, we employed structure‐based virtual screening (SBVS) to pinpoint potential CLK2 inhibitors, followed by biological validation. Firstly, a compound library was screened to identify potential inhibitors based on their docking scores. Subsequently, the selected compounds were subjected to in vitro enzymatic inhibition assays to assess their activities, and their specificity against other kinases was meticulously confirmed. Furthermore, we conducted cell‐based assays to evaluate the functional efficacy of the identified CLK2 inhibitor, including those for cell proliferation, survival, and expressions of RNA splicing factors. The comprehensive analysis of these results led to the discovery of a promising novel CLK2 inhibitor with potential applications in treating TNBC.

## 2 RESULTS

### 2.1 Identification and validation of CLK2 inhibitors

**FIGURE 1:**
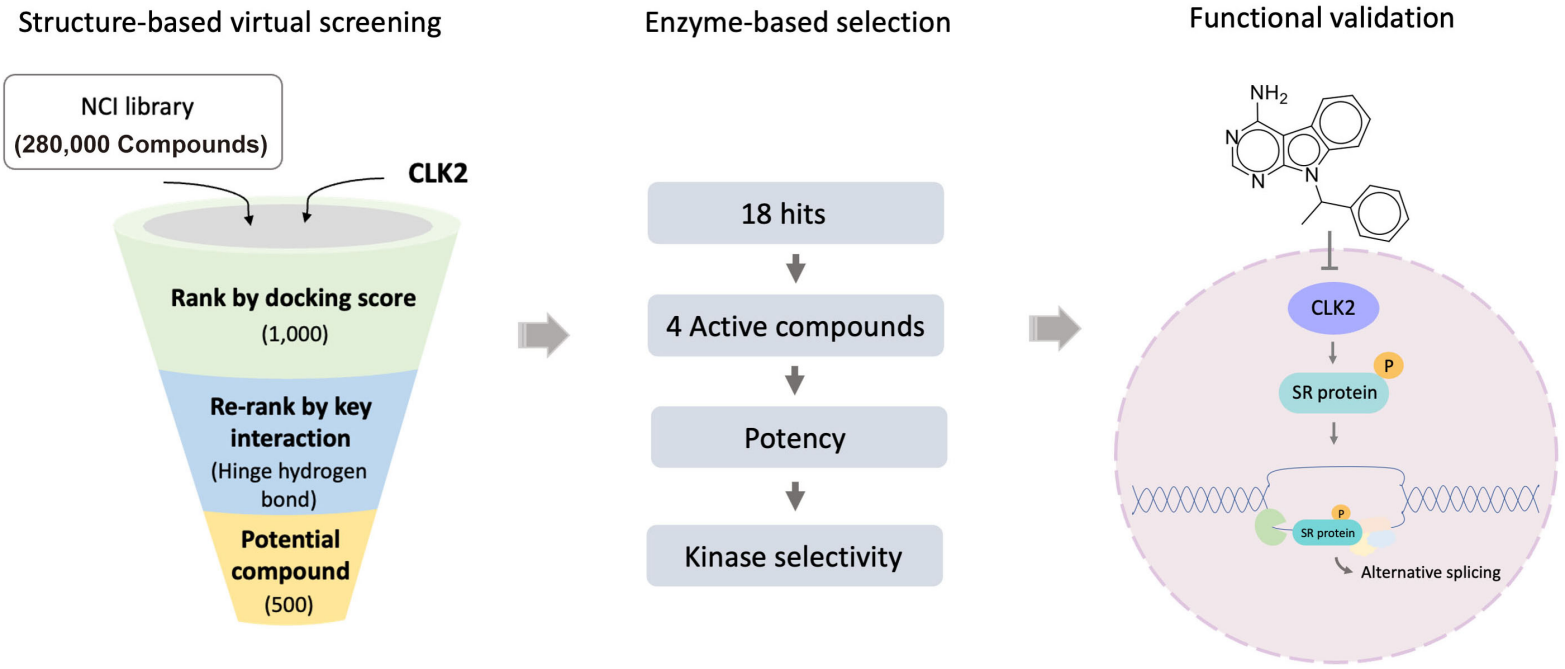
Workflow of the study. An SBVS campaign was performed using compounds from the NCI library. Their docking scores and hinge interactions were used to filter compounds. Selected compounds were validated using enzymatic assays, confirming them to be CLK2 inhibitors. Further testing confirmed CLK2 inhibition and its effects on breast cancer cells.

Recognizing the significance of CLK2 in cancers, we initiated a SBVS campaign to identify potential CLK2 inhibitors. A flow chart of this study is shown in Figure 1. The screening library consisted of molecules from the NCI compound database. The CLK2 protein structure was obtained from the Protein Data Bank (ww PDBc, 2019). Molecules were docked into the CLK2‐binding site and ranked by their associated docking scores. The top 1000 compounds were retained. Hydrogen bonds to the hinge residues are common features of kinase inhibitors (Attwood et al., 2021). Compounds lacking this interaction were removed, leaving roughly 500 compounds for selection. The remaining molecules were selected based on their docking pose and availability for testing. In total, 18 compounds were selected for validation.

Compounds were tested for CLK2 inhibitory activity at a concentration of 10 μM. A hit in this study was determined to be a compound displaying ≥50% inhibition of CLK2 activity. Of the candidate compounds, five displayed CLK2 inhibitory activity (Table 1). The most potent CLK2 inhibitor identified was 670551, which inhibited CLK activity by 96% at 10 μM. Activities of the compounds were further identified through their IC_50_ values, with 670551 again displaying the greatest potency at 619.7 nM (Table 2). Together, these results revealed an SBVS campaign that led to the identification of a CLK2 inhibitor.

**TABLE 1:** Validation of selected compounds.

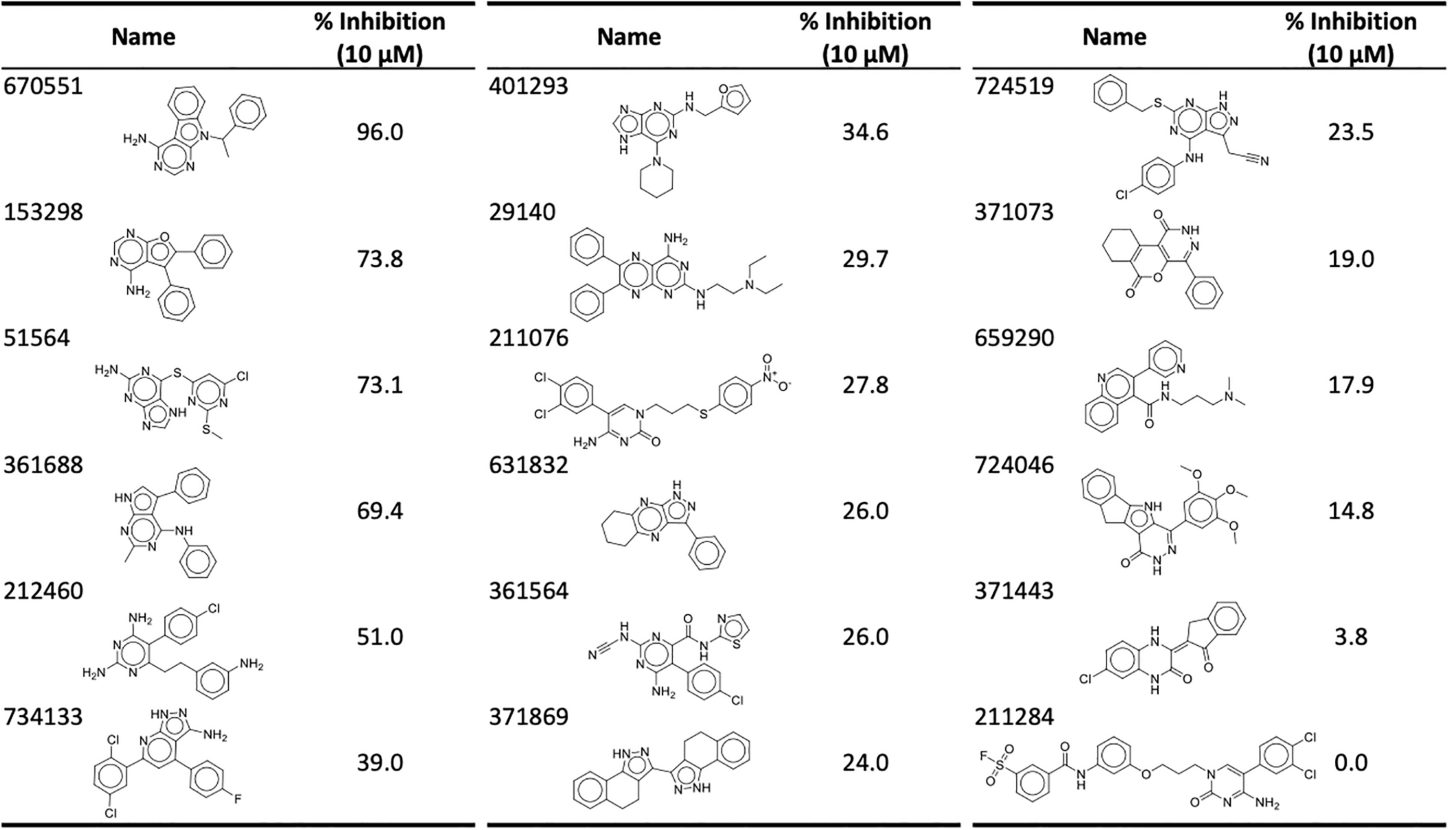

**TABLE 2:** Values of the 50% inhibitory concentration (IC_50_) of CLK2 compounds.

Compound	CLK2 IC_50_ (nM)
670551	619.7
153298	2245.0
51564	1331.8
361688	1775.6

### 2.2 Protein–ligand interaction analysis of hit compounds

**FIGURE 2:**
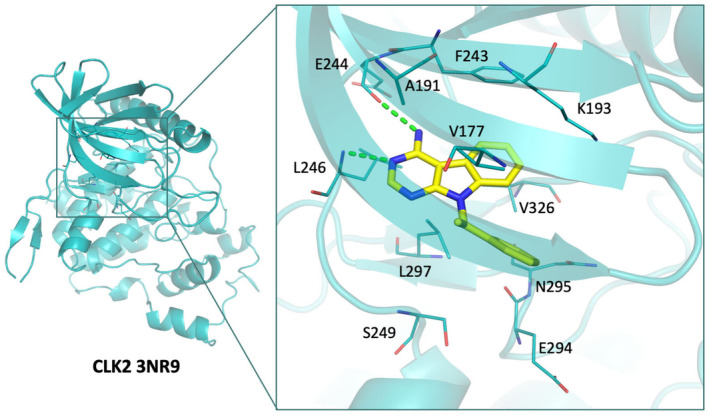
Docking pose of compound 670551. Compound 670551 (yellow) shows favorable occupation of the CLK2‐binding site (blue), such as hydrogen bonds to hinge residues. The CLK2 protein is rendered as a cartoon, and interacting binding site residues are rendered as lines. Hydrogen bonds are rendered as green dashed lines.

To further identify mechanistic reasons for CLK2 inhibition, we performed an interaction analysis of the identified inhibitor. As the most potent compound, protein–compound interactions of 670551 were analyzed (Figure 2). The main scaffold of 670551 consists of two large ringed structures. The indolo[2,3‐d]pyrimidine ring structure contains an amino group at the 4′ position. This ring structure forms two hydrogen bonds to residues L246 and E244. These residues comprise the hinge loop, and hydrogen bonds to these residues are common features that anchor inhibitors within the kinase ATP‐binding site (Attwood et al., 2021). This hydrogen bond pattern was observed in a previous analysis of kinase hinge binding scaffolds (Xing et al., 2015). Hydrophobic interactions sandwich the indolo[2,3‐d]pyrimidine ring and include residues A191, K193, F243, L246, L297, and V326. The second ringed structure consists of a methyl benzene moiety that extends into an adjacent pocket to the hinge residue region. Due to its methyl group, hydrophobic interactions were observed with residues L169 and V177. The resulting pose showed that compound 670551 can form favorable interactions with CLK2‐binding site residues.

### 2.3 Selectivity evaluation of compound 670551

**FIGURE 3:**
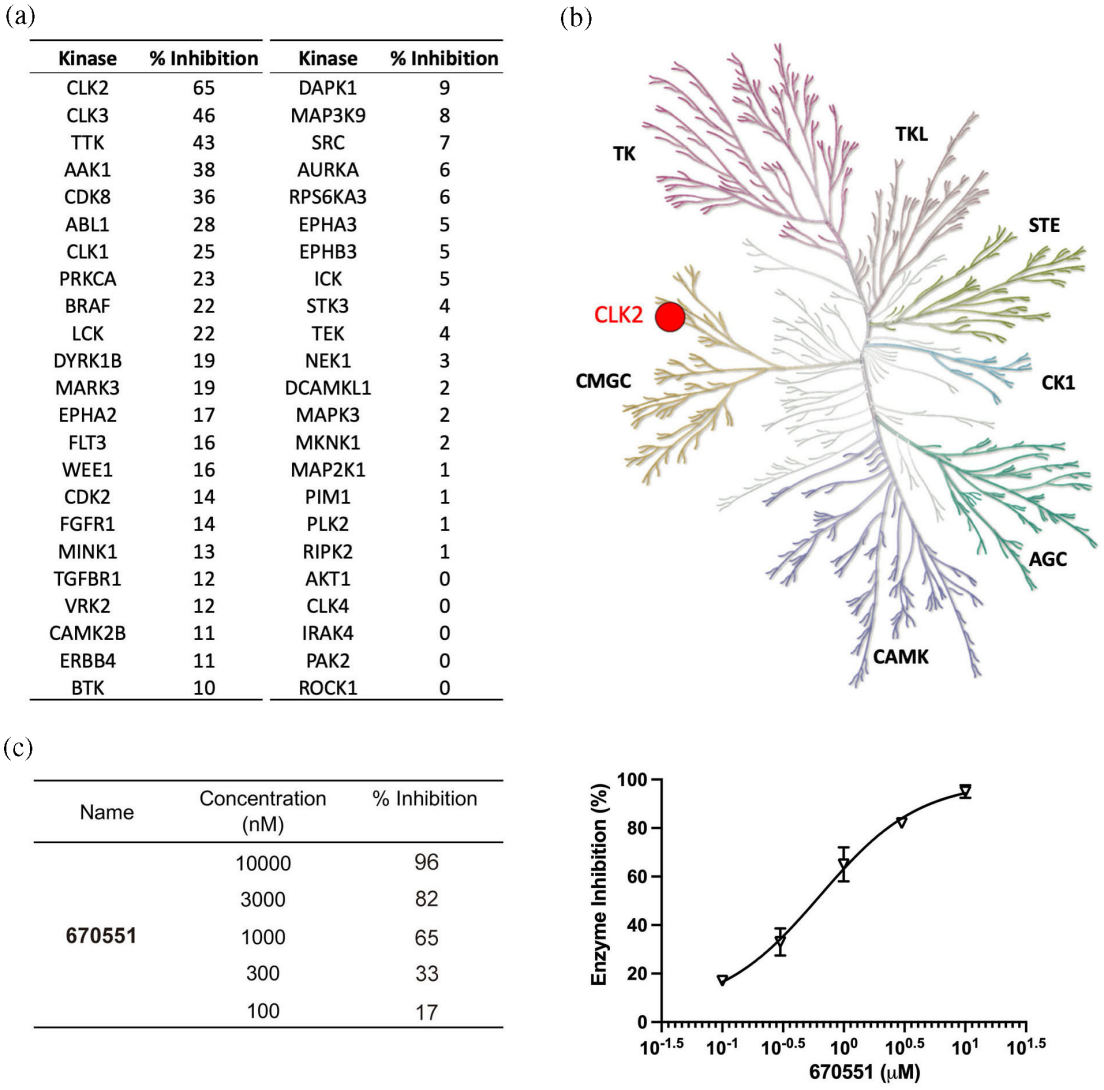
The kinase selectivity of compound 670551. (a) Compound 670551 (1 μM) was assessed against a spectrum of 46 kinases in the human KinMap. (b) Only the CLK2 inhibition percentage exceeded 50% among all of these kinases. Inhibitory effects above 50% is represented by red dot. (c) The inhibitory effect of 670551 against CLK2 at the indicated concentration.

The development of CLK2 inhibitors currently faces challenges regarding selectivity for CLK2, which may lead to adverse effects in clinical applications. To verify the selectivity of compound 670551, we tested it against approximately 40 kinases which covered seven major groups of human protein kinases. Results showed that among these kinases, only CLK2 had an inhibition percentage above 50% (65%) when exposed to 1 μM of compound 670551 (Figure 3). We further used a biochemical kinase assay to evaluate the effect of 670551 against CLK2 activity at different concentration. It showed that 670551 dose‐dependently inhibited the activity of CLK2 with IC_50_ = 619.7 nM. This evidence confirms that compound 670551 is a highly selective CLK2 inhibitor.

### 2.4 Compound 670551 inhibits TNBC cell growth and survival

**FIGURE 4:**
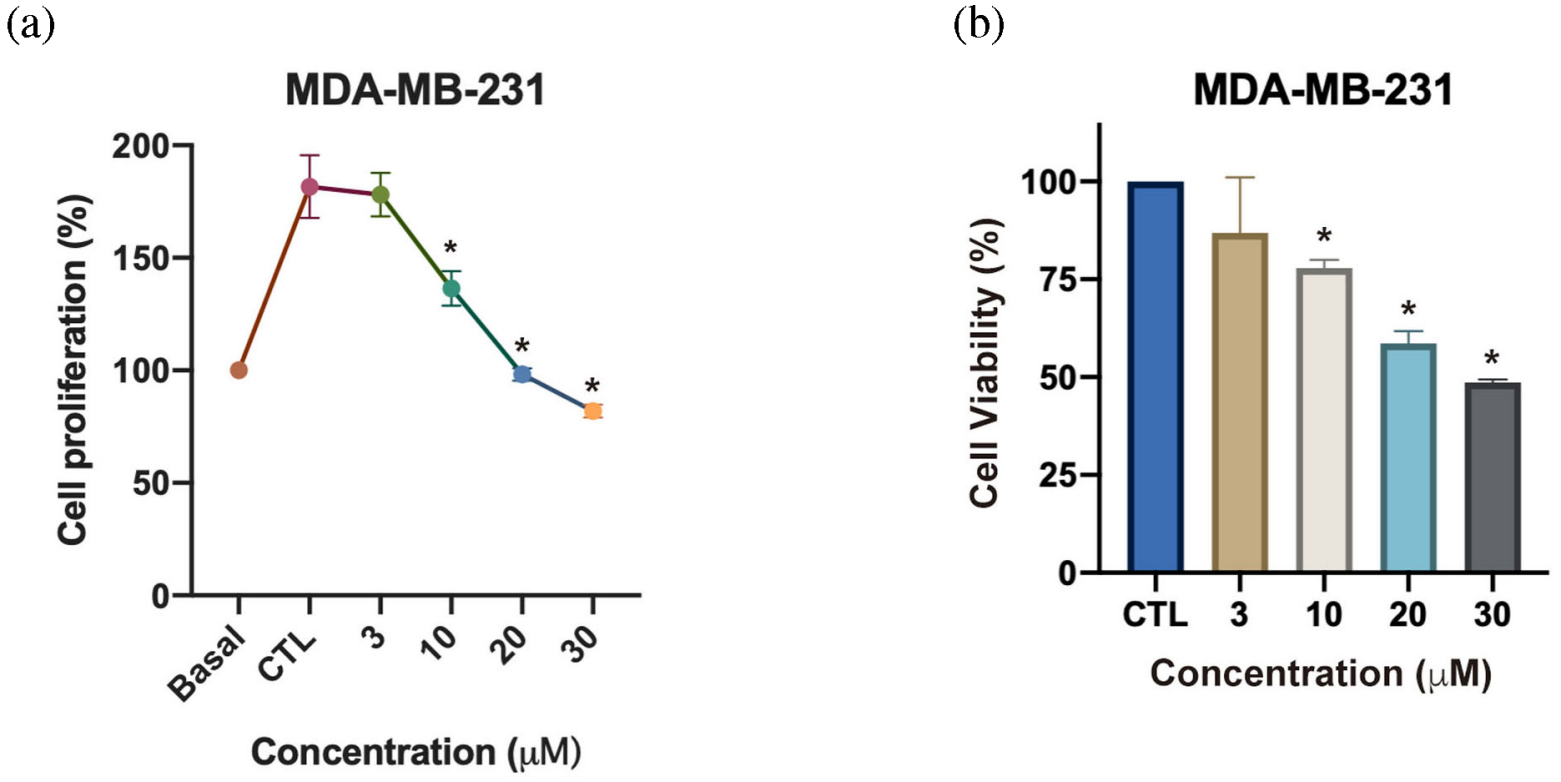
Antiproliferative and anti‐survival effects of the CLK2 inhibitor. The MDA‐MB‐231 triple‐negative breast cancer cell line was treated with different concentrations of compound 670551 for 72 h. (a) The proliferation effect was determined by an SRB assay. The cell count before treatment with DMSO or 670551 (0 h) was defined as the basal level. The cell count after 72 h of treatment with DMSO was designated as the control group. (b) Cell survival was assessed by an MTT assay. Results are based on at least three independent experiments. *p < 0.05 compared to the control group.

To validate the in vitro anticancer effect of compounds targeting CLK2, MTT and SRB assays were, respectively, used to determine cell survival and growth. The TNBC MDA‐MB‐231 cell line was treated with compound 670551 for 72 h. Results showed that the growth of MDA‐MB‐231 was dramatically suppressed in a dose‐dependent manner, and at 30 μM, the cell number decreased to the same level as the basal level (Figure 4a). Additionally, compound 670551 dose‐dependently decreased cell survival (Figure 4b). Compound 670551, a CLK2 inhibitor, collectively inhibited cell growth and survival in vitro.

### 2.5 CLK2 inhibitor downregulates serine/arginine‐rich splicing factor (SRSF) phosphorylation and induces cell apoptosis

**FIGURE 5:**
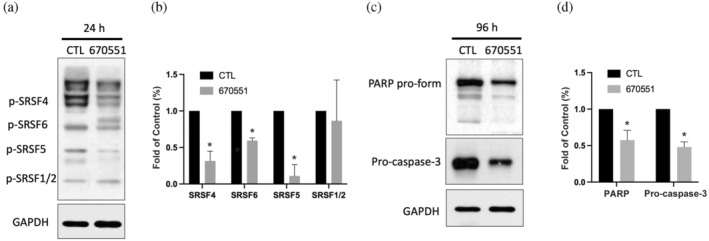
Biological effects of the CLK2 inhibitor in TNBC. MDA‐MB‐231 cells were treated with compound 670551 at a concentration of 60 μM for (a) 24 h to detect the CLK2 downstream target, serine/arginine‐rich splicing factor (SRSF), and for (c) 96 h to examine expressions of the pro‐forms of PARP and caspase‐3. (b, d) Quantitative results from (a) and (c), respectively. Data shown are results of at least three independent experiments. *p < 0.05 compared to the control group.

CLK2 was identified as a key regulator of mRNA AS through the phosphorylation of SRSF proteins. To assess the biological impact of the identified compound, we examined the phosphorylation levels of SRSF proteins. Following a 24‐h treatment of cells with compound 670551, a substantial reduction in the phosphorylation levels of SRSF4, SRSF5, and SRSF6 was observed. This finding confirms that compound 670551 functions as a potent CLK2 inhibitor. (Figure 5a, b). Previous studies have shown that pharmacological inhibition of CLK2 activity significantly induced apoptosis, contributing to its potent antitumor effects [22]. Long‐term treatment with compound 670551 led to a decrease in the pro‐forms of poly (ADP ribose) polymerase (PARP) and caspase‐3, indicating its ability to activate the apoptotic pathway in TNBC cells (Figure 5c, d). These findings strongly imply that CLK2 serves as a functional inhibitor and can be considered as a novel therapeutic approach for TNBC.

## 3 DISCUSSION

This study identified a potent CLK2 kinase inhibitor, compound 670551, using an SBVS approach. This inhibitor demonstrated significant CLK2 kinase inhibitory activity, with an IC_50_ value of 619.7 nM and high selectivity. Compound 670551 markedly impaired the survival and growth of TNBC cells. It significantly reduced essential RNA splicing proteins (SR proteins). Additionally, it promoted TNBC cell apoptosis, as indicated by the downregulation of procaspase‐3 and the pro‐form of PARP. Consequently, compound 670551 represents a promising therapeutic strategy for combating TNBC.

AS has a profound impact on human pathogenesis, including tumor progression and neurodegenerative diseases (Jain et al., 2014), and promoting the development of CLK inhibitors has been suggested. Currently, several CLK or CLK2 inhibitors are being investigated in clinical trials for their potential use in treating various conditions such as degenerative diseases, osteoarthritis, cancers, psoriasis, scleroderma, and tendinitis (Qin et al., 2021). There are five CLK inhibitors currently under clinical trials: three are pan‐CLK inhibitors (SM09419, CTX‐712, and SM08502), and two are CLK2 inhibitors (SM04690 and SM04755). The core structure of the two selective CLK2 inhibitors is indazole (Qin et al., 2021). IC_50_ values of these indazole‐based CLK2 inhibitors were 7.8 and 0.8 nM, respectively. Contrarily, the scaffold of the compound we discovered, 670511, is comprised of two rings: an indolo[2,3‐d]pyrimidine ring structure with an amino group at the 4′ position and a methyl benzene moiety. The indolo[2,3‐d]pyrimidine ring forms two hydrogen bonds to residues Leu246 and Glu244, anchoring the compound to the ATP‐binding site. The other ring, methyl benzene moiety, has hydrophobic interactions with residues Leu169 and Val177 (Figure 2). Due to the conserved structure of kinases, the targeting of their ATP binding sites with small molecules often encounters selectivity challenges. Since CLK2 belongs to CMGC family, inhibitors designed to target CLK2 might inadvertently affect other family members, such as dual specificity tyrosine‐phosphorylation‐regulated kinase 1A (DYRK1A) (Deshmukh et al., 2021). DYRK1A plays a crucial role in neurogenesis, and dysregulation of DYRK1A results in severe neurodegenerative diseases such as Down syndrome or DYRK1A‐haploinsufficiency syndrome (Arbones et al., 2019). Therefore, maintaining precise control over DYRK1A activity is essential. It showed that the differential inhibitory activity of SM04690 between CLK2 and DYRK1A is only two‐fold, indicating a lack of selectivity. In comparison, compound 670551 selective binds to CLK2 without significantly inhibiting DYRK1A (Figure 3). Additionally, 670551 inhibited CLK3 with a 46% inhibition at a concentration of 1 μM. Similar to CLK2, CLK3 is involved in regulating pre‐mRNA splicing via SRSF protein activation (Duncan et al., 1998) and has been identified as an oncogenic kinase (Zhou et al., 2020). Previous research suggests that simultaneous inhibition of both CLK2 and CLK3 results in a more pronounced anti‐tumor effect, highlighting their significance as potential therapeutic targets (Tam et al., 2020). Threonine tyrosine kinase (TTK) is another potential target of 670551, showing an inhibitory activity of 43% at 1 μM. TTK, or Monopolar Spindle 1 (Mps1), is a dual‐specificity kinase essential for cell cycle regulation, especially in the mitotic checkpoint. It is highly expressed in rapidly dividing tissues like the testis and thymus, highlighting its crucial role in cell division and proliferation (Mills et al., 1992). Therefore, inhibition of TTK may interfere mitotic machinery, leading to chromosomal instability. However, TTK is also prevalently expressed in cancerous tissues (Zeng et al., 2023). TTK inhibition may provide efficacy in treating TNBC. Dual inhibition of TTK and CLK has been found effectively inhibited tumor growth by affecting cancer cell mitosis and RNA splicing (Riggs et al., 2017). Collectively, our findings suggest that 670551, with its primary targeting of CLK2, represents a promising candidate for further drug development.

One important role of CLKs is to phosphorylate the SR protein, SRSF 1–12, which modulates the spliceosome molecular machinery (Nayler et al., 1997). SR proteins serve as a link in CLK‐regulated mRNA splicing. These proteins are primarily found in the nucleus; certain types, like SRSF1, SRSF3, and SRSF7, can shuttle between the nucleus and cytoplasm (Cáceres et al., 1998). Since SRSFs play key roles in regulating mRNA splicing, their dysregulation can result in abnormal biological functions which may be related to tumorigenesis (Zheng et al., 2020). A comprehensive analysis of AS patterns in BC using The Cancer Genome Atlas (TCGA) database has uncovered that TNBC exhibits a higher number of AS events compared to other subtypes such as luminal A, luminal B, or HER2‐positive breast cancers. Moreover, a significant expression of various splicing factors, including SR proteins, SR‐related kinases, and hnRNPs, is observed in TNBC samples compared to non‐TNBC cases (Ke et al., 2018). Among these, the splicing factor SRSF1 is noted for its involvement in numerous AS events in breast cancer. It is responsible for the inclusion of exon 9 in *CASC4*, promoting tumor proliferation and reducing apoptosis (Anczukow et al., 2015). Another study highlights that SRSF1 also regulates the inclusion of exon 3 in PTPMT1, which contributes to tumorigenesis via the AKT/MYC signaling pathway (Du et al., 2021). The knockdown of TDP43 disrupts its interaction with SRSF3, leading to a reduction in metastatic and proliferative capacities in TNBC (Ke et al., 2018). Despite evidence pointing the critical role of SRSF in tumor pathogenesis, the potential therapeutic impact of targeting SRSF in TNBC remains unclear. In this study, we found that the CLK2 inhibitor, 670551, statistically inhibited SRSF4 and SRSF6 (Figure 5a, b). SRSF6 was reported to be significantly amplified in breast, lung, ovarian, and colon cancers, and promotes oncogenic splicing and further controls cell proliferation, metastasis, and drug resistance (Adhikary & Eilers, 2005; She et al., 2021). Additionally, not all splicing factors influence tumorigenesis in BC; a previous study found that SRSF4 and SRSF6 were involved in acinar morphogenesis and promoted cell proliferation and invasion (Park et al., 2019). SRSF4 also contributed to cisplatin‐induced cell death in BC cells (Gabriel et al., 2015). Furthermore, CLK2 was observed to be overexpressed in BC, and deletion of CLK2 reduced tumorigenesis. Therefore, we propose that this is why the CLK2 inhibitor, which alters SRSF4 and SRSF6, can induce apoptosis in BC cells. This is evidenced by decreases in procaspase‐3 and PARP (Figure 5c, d).

To further evaluate the potential of compound 670551 in cancer treatment, we utilized ProTox‐II to further study the safety profile of the compound. ProTox‐II is a web server designed to predict the toxicity of chemicals, classifying them based on the median lethal dose (LD_50_). Class I is the most lethal, with an LD_50_ of <5 mg/kg, while class VI is the safest category, with an LD_50_ of >5000 mg/kg. The prediction showed that compound 670551 belongs to class IV, with a predicted LD_50_ of 500 mg/kg (Table 3). The Toxicology in the 21st Century (Tox21) program provides a library of 10,000 chemical data evaluated against 12 crucial biological pathways that regulate human development, metabolic homeostasis, and reproduction (Table 4). These pathways are classified as the nuclear receptor (NR) pathway and stress response (SR) pathway. The ProTox‐II server also offers predictions for these pathways based on the library provided (Banerjee et al., 2018). Our findings indicated that compound 670551 was inactive in most pathways except for the aryl hydrocarbon receptor (AhR) pathway. However, the probability of this prediction was only 0.67, suggesting further confirmation is required in the future. Overall, based on the predicted assessment, the CLK2 inhibitor, 670551, is considered a safe compound suitable for further development.

**TABLE 3:** Predicted 50% lethal dose (LD_50_; mg/kg) of compound 670551.

Compound	Predicted LD_50_ (mg/kg)	Predicted toxicity class	Prediction accuracy
670551	500	4	68.07

**TABLE 4:** Predicted toxicity effects on the Tox21 nuclear receptor signaling pathway and stress response pathway.

Classification	Target	Prediction	Probability
Tox21‐Nuclear receptor signaling pathways	Aryl hydrocarbon Receptor (AhR)	Active	0.67
	Androgen Receptor (AR)	Inactive	0.99
	Androgen Receptor Ligand Binding Domain (AR‐LBD)	Inactive	0.84
	Aromatase	Inactive	0.75
	Estrogen Receptor Alpha (ER)	Inactive	0.77
	Estrogen Receptor Ligand Binding Domain (ER‐LDB)	Inactive	0.96
	Peroxisome Proliferator‐Activated Receptor Gamma	Inactive	0.97
Tox21‐Stess response pathways	Nuclear factor (erythroid‐derived 2)‐like 2/antioxidant responsive element (nrf2/ARE)	Inactive	0.85
	Heat shock factor response element (HSE)	Inactive	0.85
	Mitochondria Membrane Potential (MMP)	Inactive	0.71
	Phosphoprotein (Tumor Suppressor) p53	Inactive	0.65
	ATPase family AAA domain‐containing protein 5 (ATAD5)	Inactive	0.88

In summary, our study employed a Structure‐Based Virtual Screening (SBVS) method to uncover a novel CLK2 inhibitor. This inhibitor, compound 670551, displayed strong and selective binding to CLK2 and showed activity against serine/threonine and tyrosine‐containing substrates, including SR proteins. Additionally, functional assays validated the ability of this CLK2 inhibitor to induce apoptosis in triple‐negative breast cancer (TNBC) cells. Thus, targeting CLK2 emerges as an innovative strategy for TNBC treatment.

## 4 MATERIALS AND METHODS

### 4.1 Compound preparation and molecular docking

The compound library was first prepared using Pipeline Pilot (BIOVIA, Dassault Systèmes, 2021). The National Cancer Institute (NCI) compound database containing ~280,000 compounds was selected for screening. Compounds in the library that are likely to be poor candidates were removed using a high‐throughput screening (HTS) filter. This includes molecules containing non‐organic atom types, reactive structures, and compounds with a molecular weight of >150. The remaining molecules were then filtered for drug‐like features and the presence of a Pan Assay Interference Structure (PAINS) (Baell & Nissink, 2018; Benet et al., 2016).

The remaining molecules were molecularly docked using the Schrödinger Maestro software suite (Schrödinger Release 2023‐2, 2021). Both the compound library and CLK2 protein structure were prepared using Maestro. The CLK2 crystal structure (PDB ID: 3NR9) was prepared by removing water molecules, assigning bond orders, and adding missing hydrogen atoms. The binding site was centered on the co‐crystal ligand. Docking was performed using Glide (Friesner et al., 2004). Protein–ligand interactions between the CLK2‐binding site and docked molecules were obtained using Pipeline Pilot. The docking score ranked the docking poses with the top 1000 molecules selected. Further filtering was performed based on the presence of hydrogen bonds to the CLK2 hinge residue (Attwood et al., 2021). Finally, potential inhibitors were selected and purchased depending on availability. Obtained molecules were then tested for CLK2 inhibitory activity.

### 4.2 Protein kinase activity assay

The in vitro enzymatic activity of the CLK2 kinase was outsourced to Thermo Fisher Scientific (Waltham, MA) for testing (www.thermofisher.com/selectscreen). Thermo Fisher Scientific uses Z'LYTE technology, which is based on fluorescence resonance energy transfer (FRET), to conduct testing. Test compounds were screened in 1% dimethyl sulfoxide (DMSO). The test compound, peptide/kinase mixtures, and ATP solutions were placed in the kinase buffer and incubated at room temperature for 60 min. The development reagent solution was then added to the mixtures and allowed to react for 60 min. Results were determined using a fluorescence plate reader.

### 4.3 Cell culture

The MDA‐MB‐231 cell line is a highly metastatic and invasive epithelial cell line derived from human BC. It is classified as TNBC as it lacks expressions of HER2, ER, and PR. The MDA‐MB‐231 cell line was commercially obtained from the Bioresource Collection and Research Center (BCRC, Hsinchu, Taiwan) and was cultured in L‐15 medium (Thermo Fisher Scientific) supplemented with 10% fetal bovine serum and a 1% penicillin–streptomycin‐amphotericin B solution (Biological Industries, Kibbutz Beit‐Haemek, Israel) without CO_2_ at 37°C.

### 4.4 Cell viability

A 3‐(4,5‐dimethylthiazol‐2‐yl)‐2,5‐diphenyltetrazolium bromide (MTT) assay evaluated cell viability after treatment with the test compound. Cells were seeded in 96‐well plates, and once attached, cells were treated with the indicated concentrations of the test compounds for 72 h. DMSO was used as the control group. An MTT solution containing 0.5 mg MTT reagent (Sigma Chemical, St. Louis, MO, USA) in phosphate‐buffered saline (PBS) was added to the plates and incubated for 1 h. The solution was suctioned off, and DMSO was added to solubilize the purple crystals. Cell viability (or mitochondrial activity) was determined by colorimetric detection at a wavelength of 570 nm using a Beckman Coulter plate reader (Brea, CA). The amount of color produced was proportional to the number of viable cells. The 50% inhibitory concentration (IC_50_) represents half of the survival inhibition.

### 4.5 Cell proliferation

A sulforhodamine B (SRB) assay was used to measure cell proliferation. Cells were seeded in 96‐well plates, similar to the MTT assay. After attachment, cells were treated with specified conditions for 72 h. The SRB dye binds to proteins, allowing the number of cells to be evaluated. In the assay, cultured cells were fixed with 10% trichloroacetic acid (TCA), washed several times with PBS, and stained with an SRB solution (0.4% SRB in 1% acetic acid) for 15 min. Finally, the SRB dye was washed with 1% acetic acid and dried, and the bound dye was solubilized in a Trizma‐based condition (10 mM). The absorbance of the dye in the solution was measured at OD 565 nm using an enzyme‐linked immunosorbent assay (ELISA) reader (Beckman Coulter Diagnostics, Brea, CA, USA). The 50% growth inhibition (GI_50_) represents half of the growth inhibition.

### 4.6 Western blotting

Western blotting was utilized to analyze and quantify protein expressions after treatment with the test compound. Cancer cells were seeded in 25 T at a density of 6 × 10^5^ cells/plates. The following day, cells were treated with the indicated concentrations of compounds for 24 or 96 h. Cells were harvested, and pellets were lysed with RIPA lysis buffer followed by sonication. The solutions were then centrifuged at 1.3 × 10^5^ rpm for 30 min, and the supernatant was collected and quantified using a BCA protein assay kit (Thermo Fisher Scientific, Rockford, IL). An equal amount of total protein was separated by sodium dodecylsulfate polyacrylamide gel electrophoresis (SDS‐PAGE) and transferred onto a polyvinylidene difluoride (PVDF) membrane. Primary antibodies were used to identify the target protein overnight at 4°C. Membranes were washed several times with Tris‐buffered saline with 0.1% Tween20 (TBST) and incubated with corresponding secondary antibodies conjugated with horseradish peroxidase (HRP; Jackson ImmunoResearch, West Grove, PA). Finally, images were captured using an enhanced chemiluminescence detection kit (Amersham, Buckinghamshire, UK), and the quantified results were determined using ImageJ software. Primary antibodies against phosphoepitope SR proteins was purchased from Millipore (MABE50); PARP was purchased from Cell signaling (#9532); and caspase‐3 was obtained from Novus Biologicals (NBP1‐90125).

### 4.7 Statistical analysis

All biological experiments were repeated at least three times. Categorical data are presented as the mean ± SE of the mean (SEM) or as a percentage of the control group. Student's *t*‐test was used to compare statistical differences between the treatment and control groups, with *p* < 0.05 considered statistically significant.

## AUTHOR CONTRIBUTIONS

**Cheng‐Chiao Huang:** Conceptualization; methodology; writing – original draft. **Chia‐Ming Hsu:** Investigation; formal analysis. **Min‐Wu Chao:** Formal analysis; validation; writing – original draft; funding acquisition. **Kai‐Cheng Hsu:** Methodology; validation; writing – review and editing. **Tony Eight Lin:** Methodology; formal analysis. **Shih‐Chung Yen:** Methodology; formal analysis. **Huang‐Ju Tu:** Conceptualization; supervision; writing – review and editing. **Shiow‐Lin Pan:** Conceptualization; resources; project administration; funding acquisition.

## FUNDING INFORMATION

Support was received from the Ministry of Science and Technology (MOST 112‐2320‐B‐038‐052). This research was also partially supported by the Biomedical Translation Research Center, Academia Sinica (grant no. AS‐BRPT‐112‐11), and The TMU Research Center of Cancer Translational Medicine from the Higher Education Sprout Project by the Ministry of Education (MOE) in Taiwan.

## CONFLICT OF INTEREST STATEMENT

The authors declare that they have no competing interests associated with this study.
